# Cystic Fibrosis Point of Personalized Detection (CFPOPD): An Interactive Web Application

**DOI:** 10.2196/23530

**Published:** 2020-12-16

**Authors:** Christopher Wolfe, Teresa Pestian, Emrah Gecili, Weiji Su, Ruth H Keogh, John P Pestian, Michael Seid, Peter J Diggle, Assem Ziady, John Paul Clancy, Daniel H Grossoehme, Rhonda D Szczesniak, Cole Brokamp

**Affiliations:** 1 Division of Biostatistics & Epidemiology Cincinnati Children's Hospital Medical Center Cincinnati, OH United States; 2 Department of Mathematical Sciences University of Cincinnati Cincinnati, OH United States; 3 Department of Medical Statistics London School of Hygiene and Tropical Medicine London United Kingdom; 4 Department of Pediatrics University of Cincinnati Cincinnati, OH United States; 5 Division of Biomedical Informatics Cincinnati Children’s Hospital Medical Center Cincinnati, OH United States; 6 Division of Pulmonary Medicine Cincinnati Children’s Hospital Medical Center Cincinnati, OH United States; 7 James M Anderson Center for Health Systems Excellence Cincinnati Children’s Hospital Medical Center Cincinnati, OH United States; 8 Centre for Health Informatics, Computing, and Statistics Lancaster Medical School Lancaster University Lancaster United Kingdom; 9 Health Data Research UK London United Kingdom; 10 Cystic Fibrosis Foundation Bethesda, MD United States; 11 Haslinger Family Pediatric Palliative Care Center Akron Children’s Hospital Akron, OH United States; 12 Rebecca D Considine Research Institute Akron Children’s Hospital Akron, OH United States; 13 Division of Family & Community Medicine Akron Children’s Hospital Akron, OH United States

**Keywords:** application programming interface, chronic disease, clinical decision rules, clinical decision support, medical monitoring

## Abstract

**Background:**

Despite steady gains in life expectancy, individuals with cystic fibrosis (CF) lung disease still experience rapid pulmonary decline throughout their clinical course, which can ultimately end in respiratory failure. Point-of-care tools for accurate and timely information regarding the risk of rapid decline is essential for clinical decision support.

**Objective:**

This study aims to translate a novel algorithm for earlier, more accurate prediction of rapid lung function decline in patients with CF into an interactive web-based application that can be integrated within electronic health record systems, via collaborative development with clinicians.

**Methods:**

Longitudinal clinical history, lung function measurements, and time-invariant characteristics were obtained for 30,879 patients with CF who were followed in the US Cystic Fibrosis Foundation Patient Registry (2003-2015). We iteratively developed the application using the R Shiny framework and by conducting a qualitative study with care provider focus groups (N=17).

**Results:**

A clinical conceptual model and 4 themes were identified through coded feedback from application users: (1) ambiguity in rapid decline, (2) clinical utility, (3) clinical significance, and (4) specific suggested revisions. These themes were used to revise our application to the currently released version, available online for exploration. This study has advanced the application’s potential prognostic utility for monitoring individuals with CF lung disease. Further application development will incorporate additional clinical characteristics requested by the users and also a more modular layout that can be useful for care provider and family interactions.

**Conclusions:**

Our framework for creating an interactive and visual analytics platform enables generalized development of applications to synthesize, model, and translate electronic health data, thereby enhancing clinical decision support and improving care and health outcomes for chronic diseases and disorders. A prospective implementation study is necessary to evaluate this tool’s effectiveness regarding increased communication, enhanced shared decision-making, and improved clinical outcomes for patients with CF.

## Introduction

### Background

Cystic fibrosis (CF) is a life-limiting, recessively inherited disease resulting from mutations in the cystic fibrosis transmembrane conductance regulator (CFTR) gene. Irregular functioning of the CFTR protein, which controls the transport of water and salt across epithelial cells in different organ systems, primarily affects the lungs [1]. Forced expiratory volume in 1 second (FEV_1_), expressed as a percentage of an individual’s predicted value based on normative standards for age, race, height, and sex (percent predicted FEV_1_), is a measure of airway obstruction and a primary indicator of CF disease progression, severity, and efficacy of therapeutic interventions [2]. Acute decreases in FEV_1_, clinically termed *rapid decline*, occur throughout adolescence and adulthood. Early prediction of FEV_1_ decline is critical in order to initiate preventative interventions. Tools to predict rapid decline are crucial for clinical decision support and timely intervention. Various statistical models have been proposed and applied to understand and predict CF lung function over time [3,4]. Linear mixed-effects models with random intercepts and slopes are commonly employed but are problematic because lung function data are correlated within an individual over time in a potentially more complex and nonlinear manner [5]. CF studies show that lung function decline is nonlinear and heterogeneous; using an exponential correlation structure can improve predictions of lung function decline [5,6]. We recently used a nonstationary Gaussian linear mixed-effects model [7] to predict rapid FEV_1_ decline using data from the US Cystic Fibrosis Foundation Patient Registry (CFFPR) [8]. Specifically, we applied a nonlinear model to simultaneously fit both population- and individual-level FEV_1_ decline. We used integrated Brownian motion instead of random slopes to account for longitudinal correlation in each patient’s lung function trajectory. We provided risk prediction of rapid decline in the form of predictive probabilities.

### Objective

This study’s objective was to translate our predictive algorithm into an interactive web-based graphical user interface that can be integrated with electronic health record systems and utilized by CF care providers. Over a 3-year period, we codeveloped the application with algorithm statisticians, programmers, and CF care providers. We have detailed our development process, including a multiphase study to acquire and incorporate clinician feedback, and our technical approach. The resulting application, Cystic Fibrosis Point of Personalized Detection (CFPOPD), is available online [9].

## Methods

### Application User Feedback

#### Participants

This study was conducted in the Cystic Fibrosis Care Center within the Division of Pulmonary Medicine of Cincinnati Children’s Hospital Medical Center and was approved by the Cincinnati Children’s Hospital Medical Center Institutional Review Board. Individuals involved in CF clinical care were eligible to participate; these included physicians, advanced practice nurses, social workers, dieticians, pharmacists, and respiratory therapists.

#### Procedures

Clinician feedback regarding the readability, feasibility, and perceptions of the CFPOPD application was collected in 2 phases. In the first phase, participants were encouraged to provide written feedback, drawings, and verbal comments. A semistructured interview guide was tailored to assess a given clinician’s experience in using the application. Subsequent to the initial phase, additional feedback was gathered through either individual, semistructured interviews, or focus groups. Interview guides in the second phase were revised based on previously conducted clinician focus groups and revisions to the application. Clinician feedback was recorded and transcribed by MT-STAT, a commercial medical transcription company, and it was subsequently verified for accuracy and de-identified by study staff. When discussion prompted examples of specific patients or providers were referenced, names, places, family relationships, and other potentially identifying data were removed from the transcript [10].

#### Analysis

Initial interviews were analyzed using thematic analysis [11] in which transcribed data were used to generate codes based on participant feedback and were then grouped according to the arising motifs. These resulting themes and subthemes were used to advance application development.

### Application Development

#### Data and Algorithm Development

The source of patient data used during CFPOPD development and the algorithm’s development and validation has been described in detail elsewhere [8]. Briefly, we obtained data for 30,879 patients from the US CFFPR from 2003 to 2015 to train and validate our algorithm. Our model exhibited excellent predictive accuracy. Mean absolute percentage errors for the forecasted FEV_1_ values in the validation sample for 6-month, 1-year, and 2-year intervals were within 5.6%, 6.9%, and 8.6% of patients’ actual values, respectively. CFPOPD displays data from 4847 patients from the validation sample. Data within CFPOPD were de-identified by jittering demographic and clinical measurements and reassigning a separate identifier for the purpose of application development. Patients with CF contributed data to the registry at regular clinic visits that typically occurred at least once every 3 months and during suspected pulmonary exacerbations. The algorithm requires the input of a patient’s longitudinal clinical history, including FEV_1_, the number of pulmonary exacerbations in the last year, the number of clinic visits in the past year, the presence of CF-related diabetes, the presence of chronic *Pseudomonas aeruginosa* (Pa) infection, the presence of a persistent methicillin-resistant *Staphylococcus aureus* (MRSA) infection, and their utilization of public or private insurance. Furthermore, the algorithm takes as inputs time-invariant characteristics, including age and FEV_1_ at entry, year of birth categorized into different cohorts, sex, and the number of F508del alleles.

#### Software Development

CFPOPD was built using R (version 3.6.1; R Core Team) [12] and R Shiny (version 1.4.0.2; RStudio) [13], a framework for interactive web applications and data visualization using R. Other packages used for development included emo, flexdashboard (version 0.5.1.1; RStudio), and plotly (version 4.9.2.1; Plotly) [14-16]. The software version control platform *git* was used to manage changes to the source code and implement modifications to CFPOPD functionality and features. The source code was hosted on GitHub, where multiple developers could track modifications to the source code, document software issues, and catalog major revisions through software releases. Each versioned release of the CFPOPD web application was deployed within a Docker container and stored on DockerHub to ensure a reproducible and automated workflow. A public version of the application suitable for interactive exploration is hosted online [9]. This paper describes version 7.1 of the software application.

## Results

### Initial Application Development

**Figure 1:**
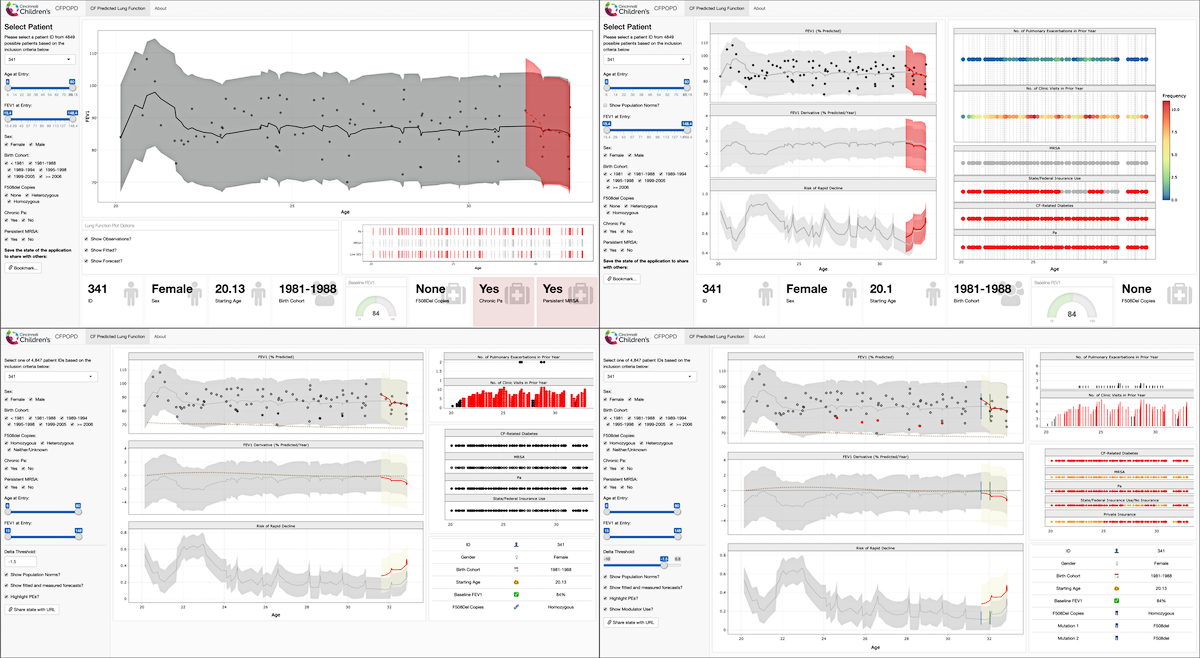
Progression of Cystic Fibrosis Point of Personalized Detection (CFPOPD) across multiple versioned releases. From versions 1 (top left) to 3 (top right), additional pulmonary function plots for the rate of forced expiratory volume in 1 second (FEV1) change and the risk of rapid decline was added. In version 5 (bottom left), users were given the ability to adjust the delta threshold to calculate the risk of rapid decline, and covariate information was moved to a table in the farthest right panel rather than a banner at the bottom of the application screen. The addition of a checkbox to visualize the initiation of modulator use was a key feature in version 7 (bottom right).

The progression of our application development is depicted in Figure 1 and shows screenshots of 4 CFPOPD versions (versions 1, 3, 5, and 7.1) in which significant revisions were implemented. Preliminary clinician feedback from CF chart and data conferences provided a blueprint for a bootstrap layout and structure, which was developed during the first 3 versions of CFPOPD [17]. The underlying layout and structure from CFPOPD (version 3) prior to formal clinician feedback remain the same in the current version, 7.1 (Figure 1). Clinician participants formally reviewed versions 3 and 7.1, and a subset of participants commented on intermittent updates to CFPOPD.

**Figure 2:**
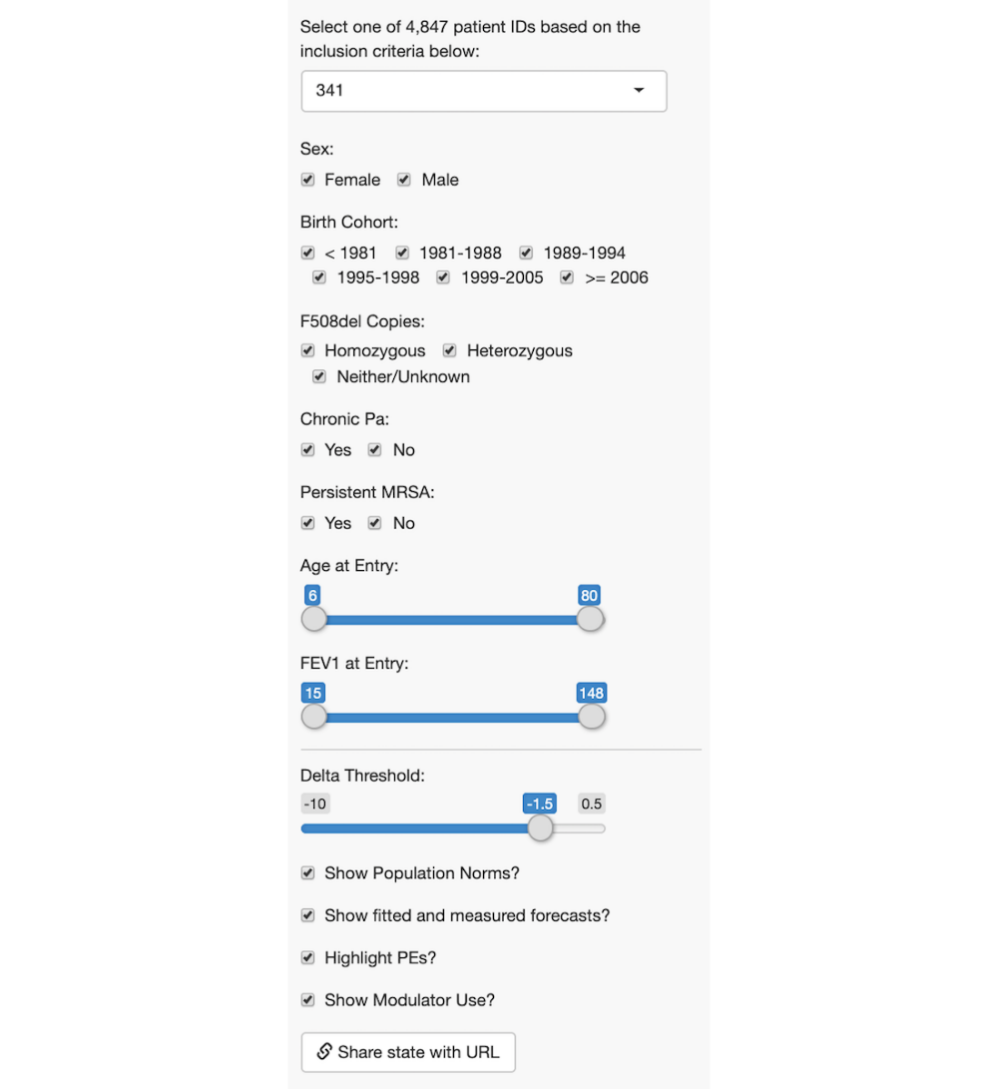
Leftmost panel of Cystic Fibrosis Point of Personalized Detection (CFPOPD). The drop-down menu shows patient 341 has been selected. Users can subset the patient sample by toggling options for sex, birth cohort, genotype (F508del copies), Pseudomonas aeruginosa (Pa) and Staphylococcus aureus (MRSA) infections, and forced expiratory volume in 1 second (FEV1) and age at entry into the US Cystic Fibrosis Foundation Patient Registry. A slider rule allows a user to select a delta threshold that is clinically relevant to a specific patient. Checkboxes allow users to select what data is viewable in the pulmonary function plots [ie, population norms, fitted and forecasted values, pulmonary exacerbations (PEs), and modulator use]. In the pictured instance, all subset and data viewing options have been selected.

The leftmost sidebar of the application includes filtering options to enable a clinician to subset the data based on model covariates and other patient-level characteristics (Figure 2). Users can select a patient to explore via a drop-down list of identification numbers. Patient data can also be filtered by toggling a sidebar checkbox and slider features for patient age at entry (coded as the first record available in the CFFPR registry data), FEV_1_ at entry into the registry, patient sex, birth cohort group, F508del copies, chronic Pa, and persistent MRSA. The list of patient identification numbers is conditional on which features are selected and the available data. For example, if the user alters the minimum value for age at entry to 16 years of age, only patients 16 years of age or older will be available for selection. Similarly, a text box above the drop-down list displays changes dynamically and displays the number of patients available based on the selected filters.

**Figure 3:**
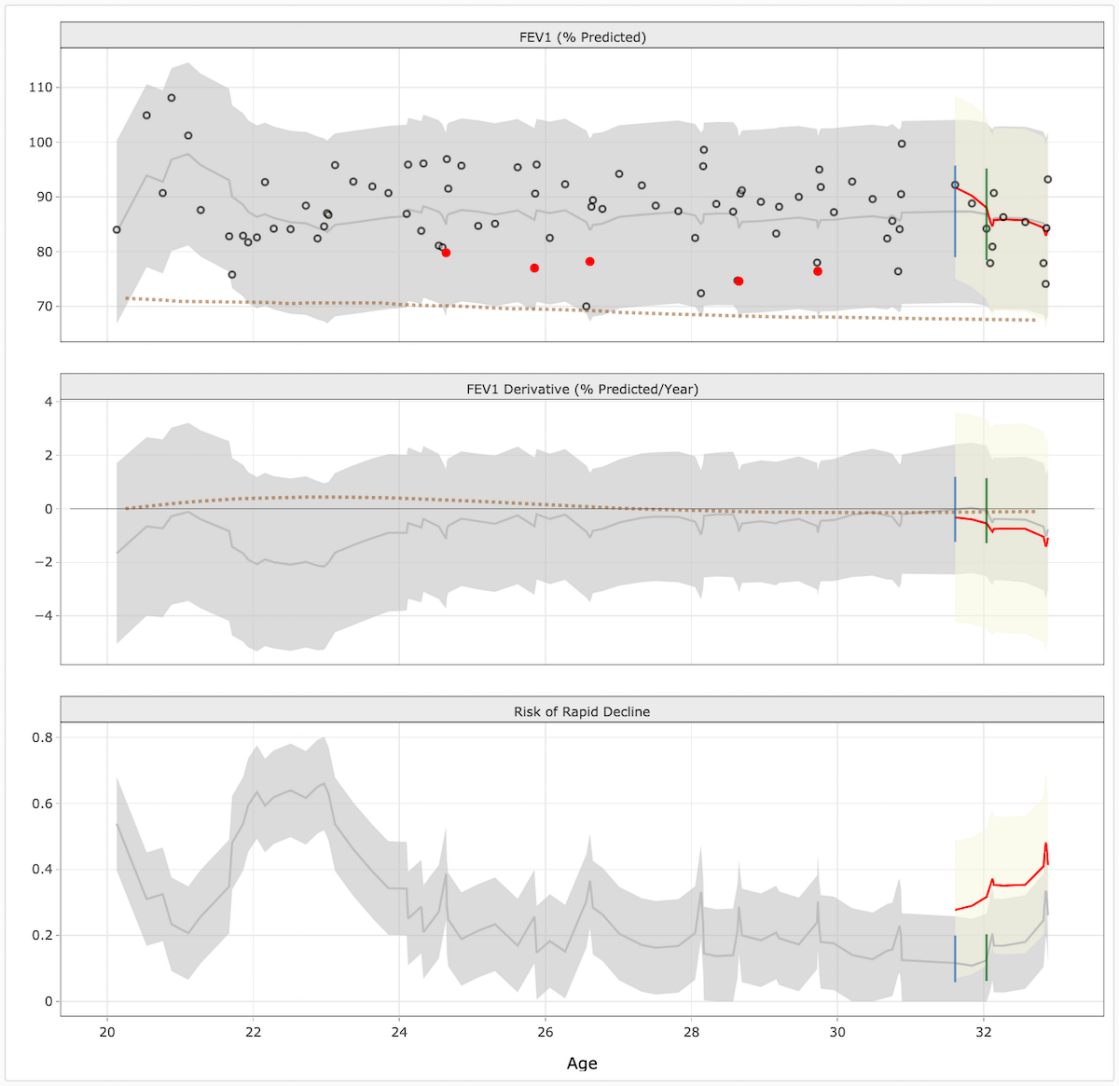
Middle panel of Cystic Fibrosis Point of Personalized Detection (CFPOPD). The 3 plots show pulmonary function data from patient 341. The top plot displays the patient’s % predicted forced expiratory volume in 1 second (FEV1) values (circles) recorded during pulmonary function testing, as well as the patient’s fitted (gray line) and forecasted (red line) values. Pulmonary function values recorded at the time a patient experienced a pulmonary exacerbation are colored red. A dotted line shows normative data (dynamic medians) respective to the patients % predicted values and rate of change in FEV1 (middle plot). The plot shows that the patient’s rate of change in FEV1 fluctuated initially but has remained stable from ages 24 to 32 years. Compared to the overall norms, patient 341’s rate of change is analogous to other patients. Similarly, the patient’s risk of rapid decline initially fluctuated but declined and stabilized (bottom plot). All plots show that patient 341 was prescribed a modulator at 31 years of age (blue line; ivacaftor) and a second modulator at 32 years of age (green line; lumacaftor/ivacaftor).

CFPOPD has 2 main plot windows. The middle panel of our current application (Figure 3) displays pulmonary function data recorded over a patient’s years of clinical follow-up via 3 faceted plots: observed percent predicted FEV_1_ (top), predicted rate of FEV_1_ decline (middle), and predicted risk of rapid decline (bottom). Together, these 3 plots facilitate clinical interpretation of a patient’s historical and future lung function trajectory. Bands surrounding each FEV_1_ trajectory line show 95% confidence intervals to demonstrate the degree of uncertainty. Bands for fitted values are colored gray, and bands representing 2-year forecasted values are beige. For the 2-year forecasted period, we show the predictions holding this interval of data out of the model (the red trend line shown in each plot); the gray trend lines represent the predictions with the data included in the model. Both sets of trend lines were presented to clinician focus group participants in order to show model fit and transparency. In addition to filtering options, users can choose what underlying data is viewable in pulmonary function plots. In version 3, one toggle was made available that allowed users to view population norms for the FEV_1_ rate of change and observed values. Normative data is generated through dynamic medians, which are computed based on the available patient data as specified by the filtering options; this was a suggestion from the aforementioned work soliciting informal feedback at chart review and data conference sessions [17].

**Figure 4:**
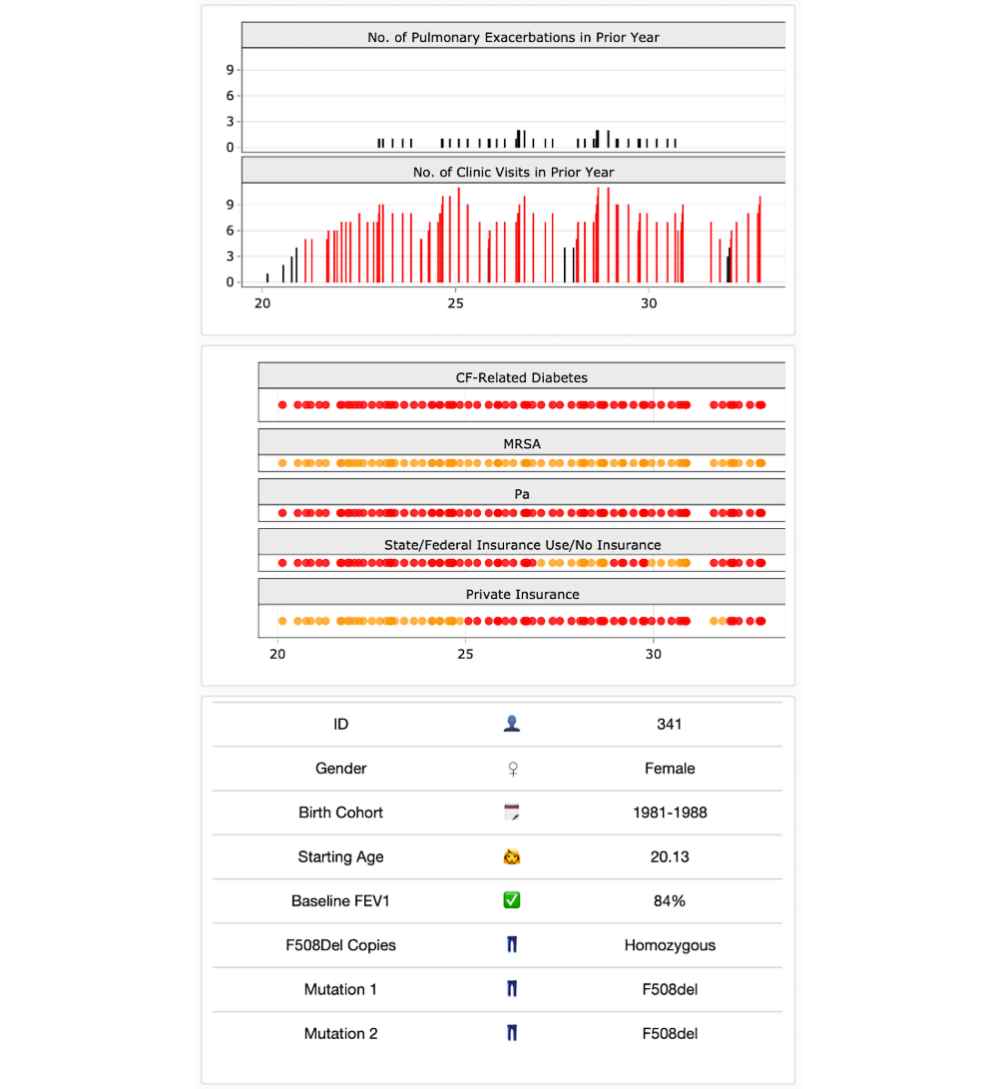
Rightmost panel of Cystic Fibrosis Point of Personalized Detection (CFPOPD). The covariate table (bottom) shows that patient 341 is female, born between 1981 and 1988, enrolled in the Cystic Fibrosis Foundation Patient Registry at age 20, had a baseline of 84% predicted forced expiratory volume in 1 second (FEV1), and is homozygous for F508del copies. The top bar plot shows that she has had few pulmonary exacerbations (PEs) but numerous clinic visits throughout her clinical history. Binary covariate plots (middle) indicate that she has been diagnosed with cystic fibrosis (CF)-related diabetes and had not developed Staphylococcus aureus (MRSA) infection but has experienced chronic Pseudomonas aeruginosa (Pa) infection since age 20. The plots for insurance type indicate that she utilized public insurance at entry and transitioned between public and private insurance, beginning at around 25 years of age.

The rightmost window in version 7.1 of CFPOPD (Figure 4) presents patient longitudinal covariate data and other disease status information such as the number of pulmonary exacerbations (denoted as “PEs” on the app) in the previous year, persistent MRSA, and CF-related diabetes. In version 3 of CFPOPD, this data was displayed using points plotted over time and colored to correspond to continuous and dichotomous variables, including the presence (red) or absence (gray) of clinical characteristics. Lastly, CFPOPD also displays time-invariant covariate information such as the selected patient’s starting age, birth cohort, sex, and number of F508del copies. In version 3, these were shown in a horizontal table below the plotting windows.

CFPOPD also features an ‘About’ tab in the top banner that describes the purpose of the application, defines application-specific terms, and instructs users how to use the data filtering and data viewing options. This section also provides a narrative of the clinical history and covariate information for an example patient (186) to illustrate CFPOPD’s utility in clinical practice.

A key feature of CFPOPD is the interactivity of pulmonary function and covariate plots. Users have the capability to zoom in and pan across a specific year in a patient’s clinical history. Faceted FEV_1_ plots are also linked. For example, if a user zooms to a specific range of ages in the bottom pulmonary function plot where a patient’s risk of rapid decline appears to change, the same period of interest will be displayed in the plots for FEV_1_ derivative and observed FEV_1_ values. The scales on the x- and y-axes also change dynamically. An additional interactive feature includes text hovering. When a user scans across the plots with the cursor, a text window will display the values of the underlying data.

### Focus Group and Conceptual Model

A total of 17 clinicians (6 attending pulmonologists, 1 nurse practitioner, and 10 pulmonary research fellows) participated in 2 formal focus group sessions (Table 1). The first session included attending physicians and a nurse practitioner, while the second session included fellows. Fellows were grouped separately from attending physicians and other standing members of the care teams, given their roles as trainees. Select participants from the attending and nurse practitioner session were followed up in individual interviews for additional feedback after CFPOPD updates were made based on the focus group. We followed up with a subset of 3 participants from the fellows’ session. Participants were chosen for follow-up based on the salience of their feedback. Prior iterations garnering feedback through CF-specific chart and data conferences consisted of 35 members across the clinical care teams.

**Table 1:** Focus group participant (clinician) characteristics (n=17).

Clinician characteristics		Women (n=9), n (%)	Men (n=8), n (%)	Total (n=17), n (%)
**Ethnicity**				
	Hispanic or Latino	0 (0%)	2 (25%)	2 (12%)
	Not Hispanic or Latino	9 (100%)	6 (75%)	15 (88%)
**Race**				
	Asian	1 (11%)	0 (0%)	1 (6%)
	Black/African American	1 (11%)	1 (12%)	2 (12%)
	White	7 (78%)	7 (88%)	14 (82%)

**Figure 5:**
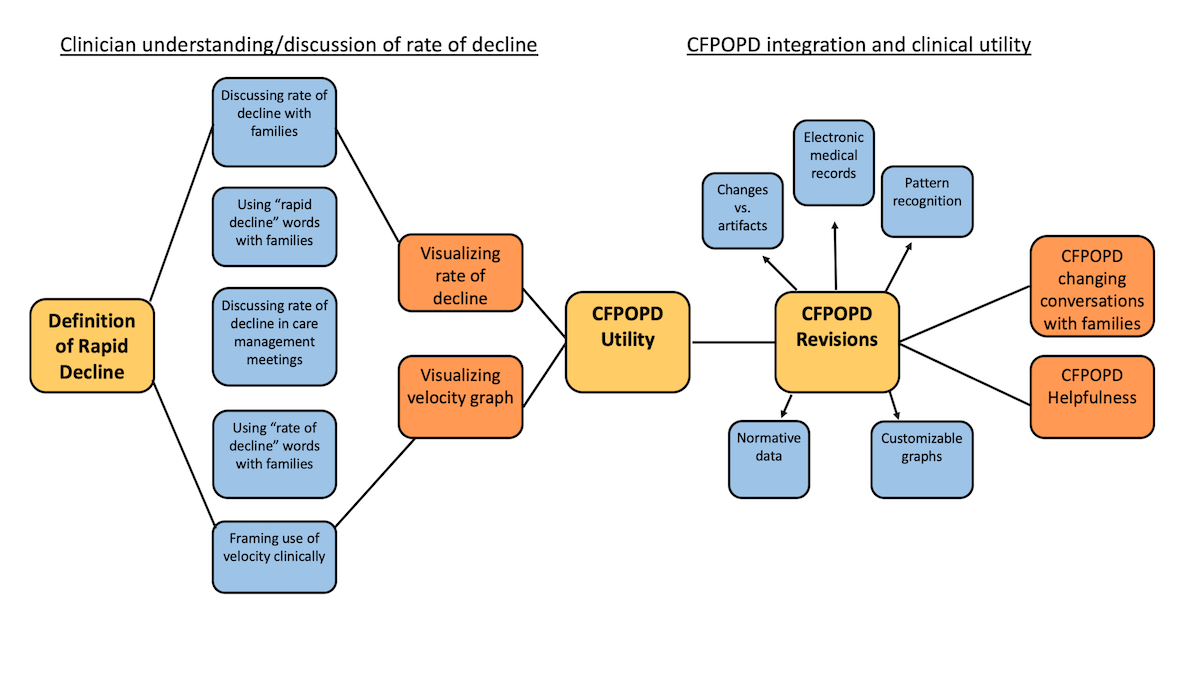
Conceptual clinical model of rapid decline. Larger yellow-shaded boxes with bold text represent key discussion points during focus group sessions. The first half of the diagram summarizes clinician understanding and discussion with other care team members and patients/families. Clinicians translated concepts of rapid decline into optimizing Cystic Fibrosis Point of Personalized Detection (CFPOPD) data visualization/monitoring capabilities. The second half represents CFPOPD integration and clinical utility. Clinicians identified challenges to the user interface and suggested revisions. The rightmost boxes represent clinician feedback on how the use of CFPOPD would change the conversations with families or otherwise be helpful.

As a result of focus group sessions, we developed a conceptual model of clinician perceptions toward rapid decline and CFPOPD integration (Figure 5). Key a priori discussion points were the definition of rapid decline, challenges to CFPOPD utility, and revisions (yellow boxes). The first discussion point illuminated how clinicians use different communication techniques with families as opposed to care teams when referring to the rate of decline. Clinicians expressed hesitation with using the phrase “rapid decline.” There was also difficulty expressed in the concept of “rate of decline” and how to conceptualize rate as velocity. Challenges to CFPOPD utility, which prompted ways to improve the application, focused on electronic health record (EHR) accessibility, distinguishing change in FEV_1_ from artifacts, and the desire to have a decision support tool that could help reveal patterns in FEV_1_ trajectories. Actionable revisions included the development of dynamic medians, which allowed for the use of normative data and customizable graphics. Participants described how CFPOPD could be used to strengthen conversations with patients and families, particularly in promoting adherence to therapies. Another identified area of potential clinical significance was its use in communicating rapid disease progression during inpatient settings, as CFPOPD could serve as a motivation to improve the clinical course or initiate antibiotic therapy to raise lung function levels. Targeted interviews prompted further CFPOPD developments.

Based on coded feedback from focus group participants and semistructured interviews, 4 primary themes were identified, providing granularity to the conceptual model in Figure 5. Each theme and corresponding illustrative quotes from focus group participants are shown in Textbox 1. Clinicians expressed uncertainty regarding the definition of rapid lung function decline (list 1, *Ambiguity*). The other 3 themes focused on the CFPOPD application’s utility, clinical significance, and suggested revisions (lists 2-4). CFPOPD facilitated the clinicians’ ability to decipher trends in a patient’s FEV_1_, recognize when a patient may be at risk of rapid FEV_1_ decline, and assist in determining the clinical impact of treatment interventions. Focus group participants stated that CFPOPD may be a useful educational tool (list 2, quotes a-d). Visualizing a patient’s clinical history assisted clinical adjudication (list 2, quotes e-f). Still, some care providers expressed concern that the CFPOPD may cause confusion in patient and family interactions (list 2, quotes g-h). Clinician feedback demonstrated that our application had the potential to advance clinical practice by facilitating decision-making, discussions with patients, and identification of rapid decline. Care providers articulated that incorporating CFPOPD into previsit planning meetings would improve point-of-care decision-making and facilitate conversations between families and the care team (list 3, quote a). Physicians stated that visualizing a patient’s risk of rapid decline may also be used as a motivator by eliciting treatment adherence (list 3, quote b). Clinicians recognized the value of CFPOPD and the capability to advance clinical practice (list 3, quotes c-f). Caution was expressed regarding its impact during inpatient visits, as it could serve as a demotivator (list 3, quote g). Provider feedback regarding revisions to CFPOPD has been critical to ensuring our instrument is translational, relevant, and impactful in clinical practice (list 4, quotes a-d).

**Emergent themes and accompanying quotes from clinician focus groups.**

Defining Rapid Decline:

- 1. Ambiguity
  - “[I am] more likely to refer to the curve in a clinical setting than to a threshold that is going to capture almost every patient.”
  - “Really hard to define.”
  - “If we were able to tweak [the definition] ‘rapid decline’…go for minimal change in lung function over time as opposed to something that might be more realistic for the patient.”

Cystic Fibrosis Point of Personalized Detection (CFPOPD) Application:

- 2. Utility
  - “Oh my gosh, it’s just what I wanted.”
  - “Yes, [I] would use graphs in preclinic meetings.”
  - “… helpful both on a sort of clinical decision-making side and describing it to families’ side.”
  - “As a fellow trainee, I feel sometimes that it’s really difficult for me to see that big picture.”
  - “Great that hovering gives you the exact numbers.”
  - “If you can show some improvement in the derivative, in the trajectory, it's more cause for optimism.”
  - “I don’t think it would be helpful at all to show a family. I think it is complicated for families; it’s complicated for me.”
  - “I like graphs in talking with families, but as a clinician, I think the only one I would feel comfortable using would be the top one.”
- 3. Clinical Significance
  - “If you have a visual representation like that, it would be substantially more helpful than me verbally saying, ‘You’re getting worse faster than we think you should.’”
  - “I would definitely show a 16-year-old who is noncompliant…‘if you don’t step it up, this is where you are going.’”
  - “10 years ago, we were just trying to look at random pieces of paper, and we never could see any of this whatsoever.”
  - “… put this in Epic.”
  - “These are things you can intervene on if you knew 5 years ago this trend was coming.”
  - “If you look at any clinical trial or any aspect of medicine, the more frequent your intervention is, the more frequent your clinic visits, the more frequent you’re ahead of this data, the better your outcomes.”
  - “… billboard of death.”
- 4. Revisions
  - “Customize threshold for rapid decline…if you want to call rapid as 3% or as 6% or 10%...you can play with that.”
  - “Add mutation classes and modulator therapy use.”
  - “Categorize continuous covariates based on clinical severity.”
  - “Different dots and colors…what’s bad and what’s steady.”

### Further Application Development

Our collaborative approach to developing CFPOPD has allowed our team of programmers to prospectively track its evolution, as shown in Figure 1. Data filters, pulmonary function data-viewing options, covariate information, coloring according to values, and icon typography were added to the application based on feedback received from clinical application users. Subsequent to clinician feedback, we implemented a feature to enable users to adjust the threshold value for percent predicted FEV_1_ loss or delta threshold, used to calculate a patient’s risk of rapid decline (Textbox 1, list 4, a). This threshold can be modified by manipulating the slider to the desired value, which ranges from -10% to 0.5% (Figure 2). The default threshold of -1.5% predicted/year was chosen previously [17].

We incorporated CF registry data on modulator use and mutation type (Textbox 1, list 4, b) through a checkbox in the left sidebar (‘Show Modulator Use?’). If a patient has been prescribed a modulator, vertical lines are shown on each pulmonary function graph at the age medication was first administered (Figure 3). When hovering over the vertical line, a window stating the name of the medication and age at administration is displayed. The names of each patient’s CFTR gene mutations were added to the covariate table (Figure 4).

Clinician feedback (Textbox 1, list 4, c-d) to categorize covariate information and assign clinical severity based on color was applied to pulmonary exacerbation and visit frequency plots. Pulmonary exacerbations are acute respiratory events that can emerge from precipitous drops in lung function. We revised the color scheme according to a categorical designation versus the continuous scale from version 3. Occurrences greater than 5 are colored red to designate an exceedance of the clinical threshold (Figure 4). In order to enhance a clinician’s ability to visualize pulmonary exacerbations and rapid decline, a checkbox option (‘Highlight PEs?’) was added to the left sidebar (Figure 2). When checked, a patient’s FEV_1_ value in the top pulmonary function plot will be colored red if a pulmonary exacerbation was observed (Figure 3).

Other CFPOPD revisions were based on informal feedback or implemented to optimize application functionality and comprehension. To maximize the space to visualize pulmonary function plots, we repositioned the covariate table underneath the covariate dot plot (Figure 4). We also increased the pixel width of the pulmonary function plots to improve readability and a checkbox that allowed users to toggle whether patient FEV_1_ values are displayed in the top pulmonary function plot (‘Show Fitted and Measured Forecasts?’). Depending on the number of spirometry results, removing FEV_1_ values from the plot may facilitate a clinician’s ability to decipher rapid decline (Figure 3). These revisions were completed under CFPOPD version 4.

We supplemented the covariate table with emojis to increase the ease of visual interpretation, implemented in version 5. Where applicable, emojis change dynamically according to the age and sex of the selected patient. The standard symbol for either male (♂️) or female (♀️) is shown to communicate the selected patient’s sex, and depending on if the patient is younger or older than 18 years of age, either a girl, boy, woman, or man emoji is shown to communicate the starting age.

Lastly, binary dot plots of the number of PEs and clinic visits a patient experienced in the previous year were modified to bar plots in version 6. In addition to colored bars indicating clinical severity, this second dimension enhances a user’s ability to visually evaluate a patient’s clinical trajectory.

## Discussion

### Principal Findings

We developed and coproduced an interactive web application designed to facilitate clinical point-of-care decision-making by predicting acute pulmonary function decline in patients with CF. We conducted focus groups with clinicians and CF care providers to garner feedback on a prototype application [17] and used this feedback to further develop the application in order to advance its utility for clinical care.

Clinicians suggested insightful and actionable CFPOPD revisions, which we incorporated over the course of 4 versioned releases. A principal revision was to add a feature enabling care providers to tailor the delta threshold according to their clinical judgment and characteristics of an individual patient. Implementing this capability was paramount to ensure CFPOPD was applicable in clinical practice. Adding this feature also manifested in a related theme regarding uncertainty toward a single clinical definition of “rapid decline.”

With the advent of modulator therapies, another requested modification was to include visualization of modulator use and descriptive text to communicate patient mutations. While numerous therapies exist to mitigate and treat acute symptoms in CF, modulator therapies act at a molecular level to restore function to CFTR protein [1,18]. By enabling care providers to detect when a patient is at risk for acute decline in pulmonary function, CFPOPD may facilitate clinical judgment and decision-making regarding the initiation of acute therapies, such as intravenous antibiotics. Previous research has shown that a treatment of acute drops in FEV_1_ using intravenous antibiotics improved long-term pulmonary function [19]. Similarly, if a patient is currently prescribed a modulator, our application allows care providers to track a patient’s lung function prospectively and assess the effectiveness of personalized treatment regimens. CFPOPD has implications for emerging studies involving patient withdrawal of maintenance therapies, given observed effectiveness for select combinations of mutations and modulators.

Technological advances in electronic data storage have transformed the management of medical records, greatly increasing the volume of data accessible to researchers, clinicians, and patients [20]. This abundance of information has yielded opportunities for novel development of interactive applications to synthesize, model, and translate EHR data [21]. Web-based applications have been employed across research and medical domains, ranging from infection management [22] to personalized mental health monitoring [23]. Likewise, others have leveraged visual analytics to translate results from complex statistical techniques used in EHR research, such as case-crossover design [24] and hierarchical clustering [25], into a comprehensible form. We sought to develop CFPOPD in order to improve point-of-care decision-making, and feedback from clinicians at our institution demonstrates our application has the potential to do so. Furthermore, clinician responses also indicate CFPOPD may promote communication and shared decision-making. Previous research indicates that participatory decision-making between physicians and their patients results in greater patient satisfaction [26]. Care providers noted that CFPOPD use may encourage adherence among patients with CF that are noncompliant, and there is empirical evidence to support this. Heisler et al [27] have shown that effective communication and shared decision-making are associated with positive diabetes self-management.

### Limitations

Although our results indicate that CFPOPD has the potential to positively impact clinical care, some feedback suggests that care provider comprehension is not universal. Additional training may be necessary before our application is fully deployed for clinical practice. Some discord existed among physicians as to whether our application would facilitate conversations between patients/families and the clinical care team, as clinicians expressed differing opinions regarding approaches to communicate a high risk of rapid decline. To accommodate this limitation, future revisions to CFPOPD could include additional options that allow care providers to customize CPOPD’s layout by selecting only plots that are relevant to the patient-provider discourse. Currently, CFPOPD is limited to existing fields available from the CFFPR data. Risk calculations are not computed in real time; rather, values are pulled from precomputed lookup tables. While our application demonstrates the predictive accuracy of our algorithm, further development is needed to integrate CFPOPD into near real-time clinical practice. Lastly, our findings are based on a single-center study (Table 1). We anticipate drawing a larger, more diverse sample of care teams in future multicenter studies assessing CFPOPD feasibility and acceptability.

### Future Work

Our future work will address CFPOPD limitations; chiefly, we will strive to implement CFOPD into an EHR system to provide “now-casting,” or near real-time statistical predictions of rapid decline. In addition to rapid decline, a similar area of extension is to calculate risk probabilities for pulmonary exacerbation onset. Recently, a data-driven definition for pulmonary exacerbation has been proposed and is being tested by the Cystic Fibrosis Learning Network [28]. Making CFPOPD available for use in clinical practice will enable assessment of its impact on clinical practice and patient outcomes. It may be desirable for patients to access their longitudinal data as well, which could potentially be made available to patients through the medical institution’s patient portal. Given emerging public health issues and a drastic increase in telehealth, integrating home spirometry into CFPOPD may become a critical priority. Combined with access to the CFPOPD application through a care provider’s patient portal, this extension could facilitate home monitoring and diagnosis of acute drops in lung function among patients with CF being clinically followed via telemedicine. The developmental framework outlined herein is capable of adaptation to different clinical markers or chronic diseases, such as diabetes and asthma, for which longitudinal tracking is valuable.

### Conclusions

We developed CFPOPD to translate a novel predictive algorithm into an interactive clinical tool to enhance early detection and forecasting of rapid pulmonary function decline in patients with CF. Our application was built through an iterative and collaborative process among programmers, statisticians, and clinicians. We have demonstrated that this framework of collaborative design between developers and end-users is successful, capable of delivering an impactful product, and may be generalized to other chronic diseases and disorders that rely on routinely collected clinical data for medical monitoring and decision-making.

## Abbreviations

CF: cystic fibrosis
CFFPR: Cystic Fibrosis Foundation Patient Registry
CFPOPD: Cystic Fibrosis Point of Personalized Detection
CFTR: cystic fibrosis transmembrane conductance regulator
EHR: electronic health record
FEV1: forced expiratory volume in 1 second
MRSA: methicillin-resistant Staphylococcus aureus
Pa: Pseudomonas aeruginosa
PEs: pulmonary exacerbations
